# Angiopoietin-like 4 based therapeutics for proteinuria and kidney disease

**DOI:** 10.3389/fphar.2014.00023

**Published:** 2014-02-25

**Authors:** Sumant S. Chugh, Camille Macé, Lionel C. Clement, Maria Del Nogal Avila, Caroline B. Marshall

**Affiliations:** Glomerular Disease Therapeutics Laboratory, Division of Nephrology, University of Alabama at BirminghamBirmingham, AL, USA

**Keywords:** diabetic nephropathy, focal and segmental glomerulosclerosis, proteinuria, angiopoietin-like 4, therapeutics, minimal change disease, *N* acetyl D mannosamine, sialic acids

## Abstract

Current drugs used to treat proteinuric disorders of the kidney have been borrowed from other branches of medicine, and are only partially effective. The discovery of a central, mechanistic role played by two different forms of the secreted glycoprotein angiopoietin-like 4 (Angptl4) in human and experimental glomerular disease has opened new treatment avenues. Localized upregulation of a hyposialylated form (lacks sialic acid residues) of Angptl4 secreted by podocytes induces the cardinal morphological and clinical manifestations of human minimal change disease, and is also being increasingly recognized as a significant contributor toward proteinuria in experimental diabetic nephropathy. Oral treatment with low doses of *N*-acetyl-D-mannosamine, a naturally occurring precursor of sialic acid, improves sialylation of Angptl4 *in vivo*, and reduces proteinuria by over 40%. By contrast, a sialylated circulating form of Angptl4, mostly secreted from skeletal muscle, heart and adipose tissue in all major primary glomerular diseases, reduces proteinuria while also causing hypertriglyceridemia. Intravenous administration of recombinant human Angptl4 mutated to avoid hypertriglyceridemia and cleavage has remarkable efficacy in reducing proteinuria by as much as 65% for 2 weeks after a single low dose. Both interventions are mechanistically relevant, utilize naturally occurring pathways, and represent new generation therapeutic agents for chronic kidney disease related to glomerular disorders.

Current therapy for kidney disease in general and kidney disease related to proteinuric disorders in specific has relied upon the use of agents borrowed from other fields. One category of agents used to treat glomerular disease have immunosuppressive properties, and include glucocorticoids, cyclophosphamide, azathioprine, chlorambucil, mycophenolate mofetil, cyclosporine, tacrolimus, and the anti-CD20 antibody. Another category contains drugs used for supportive therapy, including a variety of diuretics and agents that block the renin angiotensin system at different levels, like angiotensin converting enzyme inhibitors, angiotensin receptor blockers, spironolactone, and more recently, renin inhibitors like aliskiren. The traditional rationale behind the use of the first category of drugs was their immunosuppressive effect, but it has become clear over the past decade that many of these drugs have direct effects on resident glomerular cells (Faul et al., 2008; Clement et al., 2011). The concept of blocking the renin angiotensin system flourished in the 20th century, since at least partial efficacy in reducing proteinuria and slowing the progression of kidney disease was noted, and there were no other known pathogenic pathways that could be targeted.

## EMERGENCE OF ANGIOPOIETIN-LIKE 4 AS A THERAPEUTIC AGENT AND TARGET

**FIGURE 1:**
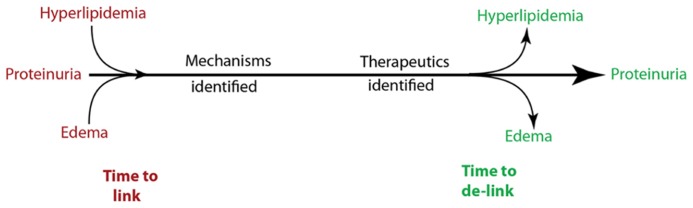
**Overall strategy for development of novel therapeutic modalities to treat proteinuria and chronic kidney disease**.

The 21st century witnessed a revolution in the identification of genes and proteins related to glomerular diseases, that can now be organized into drug targetable disease pathways. Even though these pathways are incomplete, it does not preclude the scientific community from developing new and more specific treatment strategies, if suitable end points are noted in experimental studies. The overall approach in our laboratory has been to identify a protein involved in the pathogenesis of proteinuria and at least one additional component of nephrotic syndrome (**Figure 1**). By grouping hypoalbuminemia with proteinuria and lipiduria with hyperlipidemia, we used three functional components of nephrotic syndrome for our studies: proteinuria, hyperlipidemia (hypertriglyceridemia and hypercholesterolemia), and edema. Once a gene involved in at least two of these three components was identified, its molecular pathways were dissected, and therapeutic strategies were developed specifically to reduce proteinuria without aggravating the other components of nephrotic syndrome. During discovery phase experiments (Liu et al., 2006; Clement et al., 2011) conducted in 2002 using glomeruli from highly proteinuric rats, we noted that the most highly upregulated gene out of forty differentially expressed genes fulfilled this criteria. This gene, angiopoietin-like 4 (Angptl4), had just been cloned (Kersten et al., 2000; Yoon et al., 2000) and identified as a PPAR target gene, and recombinant Angptl4 protein was shown to induce hypertriglyceridemia when injected into rodents (Yoshida et al., 2002).

**FIGURE 2:**
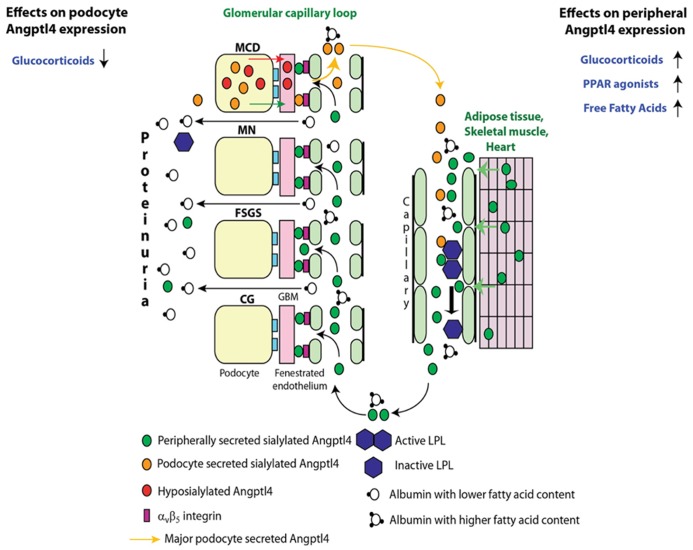
**Pathobiology of Angptl4 in nephrotic syndrome.** In minimal change disease (MCD), podocytes secrete a hyposialylated form that remains restricted to the kidney and induces proteinuria, and a sialylated form that enters the circulation. Treatment with glucocorticoids reduces podocyte Angptl4 upregulation. In all forms of primary glomerular disease like membranous nephropathy (MN), focal and segmental glomerulosclerosis (FSGS), non-HIV collapsing glomerulopathy (CG), and MCD, peripheral organs (mostly skeletal muscle, heart and adipose tissue) secrete the circulating, sialylated form of Angptl4. Treatment with glucocorticoids, PPAR agonists and free fatty acids increase peripheral organ Angptl4 secretion. Circulating Angptl4 binds to glomerular endothelial α_v_β_5_ integrin to reduce proteinuria, or inactivates endothelium bound lipoprotein lipase (LPL) in skeletal muscle, heart and adipose tissue to reduce hydrolysis of plasma triglycerides to free fatty acids (FFAs), resulting in hypertriglyceridemia. Some Angptl4 and LPL are lost in the urine. Adapted from Clement et al. (2014).

Initial studies revealed increased podocyte expression of Angptl4 in human and experimental minimal change disease (MCD), transient upregulation after the onset of proteinuria in experimental membranous nephropathy (MN), and no change in podocyte expression in non-HIV collapsing glomerulopathy (CG) and focal and segmental glomerulosclerosis (FSGS) (Clement et al., 2011). Further investigation revealed two types of Angptl4 protein in nephrotic syndrome (**Figure 2**): (a) A hyposialylated form secreted from podocytes in MCD (Clement et al., 2011), and later also noted in glomeruli of Zucker Diabetic Fatty rats (Chugh, 2011b). Conversion of this high pI hyposialylated Angptl4 to sialylated neutral pI Angptl4 *in vivo* using the sialic acid precursor *N*-acetyl-D-mannosamine (ManNAc) reduces proteinuria (Clement et al., 2011), and this improvement in proteinuria was noted in rats with MCD (Clement et al., 2011) and diabetic nephropathy (Chugh, 2011b). (b) A neutral pI sialylated form of Angptl4 is increased in the circulation of patients with MCD, MN, FSGS, and CG (Clement et al., 2014). Most of this sialylated protein is secreted from skeletal muscle, heart and adipose tissue when proteinuria reaches nephrotic range in an attempt to reduce proteinuria through glomerular endothelial binding, but also induces hypertriglyceridemia via inhibition of lipoprotein lipase (LPL). Mutant forms of this protein generated by our lab reduce proteinuria without affecting plasma triglyceride levels in nephrotic rats with FSGS and diabetic nephropathy (Clement et al., 2014).

Since circulating Angptl4 is a major molecular mediator of nephrotic syndrome due to a variety of glomerular diseases, it is an ideal candidate for a parenteral once-a-month therapeutic agent to treat proteinuric disorders that cause chronic kidney disease. On the other hand, ManNAc is orally bioavailable, and has enormous potential in low doses to treat diabetic nephropathy and as a maintenance drug to prevent MCD relapse after the first episode is treated with glucocorticoids.

## RATIONALE BEHIND SIALYLATION BASED THERAPEUTICS FOR MCD AND DIABETIC NEPHROPATHY

Our laboratory described the upregulation of podocyte secreted Angptl4 in human and experimental MCD, and developed a transgenic rat model, the NPSH2-Angptl4 rat, to replicate its effects (Clement et al., 2011). These rats develop most of the classic features of human MCD, including nephrotic range and selective proteinuria, diffuse effacement of podocyte foot processes, loss of glomerular basement membrane charge, and studies in the PAN model showed that the gene is glucocorticoid sensitive *in vivo*. Podocytes in NPHS2-Angptl4 transgenic rats, and PAN rats in the early glucocorticoid-sensitive phase overproduce a hyposialylated form of Angptl4. Treating these rats with ManNAc improves the sialylation of Angptl4 *in viv*o, and reduces proteinuria by at least 40%. By contrast, 2D gel migration pattern of podocalyxin, a sialylated structural protein expressed in podocytes, is not altered in a manner to suggest an effect on structural proteins (Clement et al., 2011). Subsequent studies show the existence of high pI hyposialylated Angptl4 in glomeruli of rats with diabetic nephropathy, and treatment of Zucker Diabetic Fatty rats with ManNAc resulted in a significant decline in proteinuria (Chugh, 2011b). These studies suggest that even though diabetic nephropathy is a multicomponent disease (i.e., has features of many primary glomerular diseases, and more), the contribution of hyposialylated Angptl4 to this disorder is prominent, and warrants therapy. Molecular pathways leading up to sustained Angptl4 upregulation in MCD, and not in other primary glomerular diseases, have not as yet been published.

**FIGURE 3:**
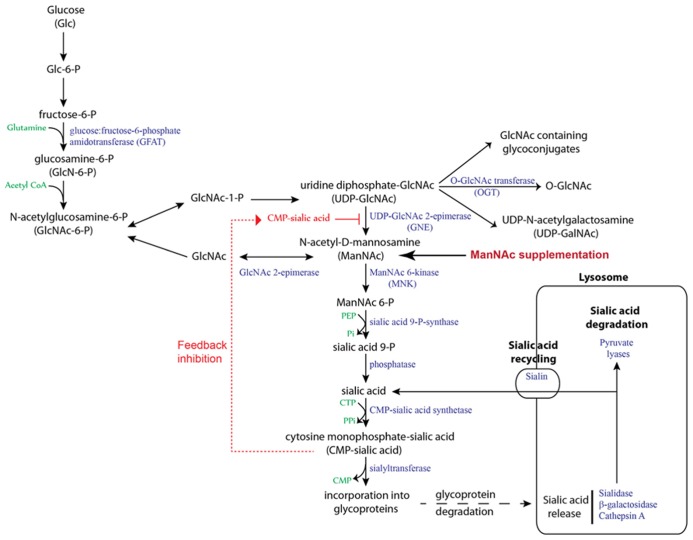
**Sialic acid biosynthesis and recycling pathway.** In humans, sialic acid is synthesized from glucose. The rate limiting step, catalyzed by GNE, is subject to feedback inhibition. ManNAc is the product of this rate limiting step, so exogenous ManNAc supplementation enters the pathway after this step. A substantial amount of sialic acid is recycled via the anion transporter sialin following lysosomal degradation of glycoproteins and glycolipids.

There are several factors that contribute toward the generation of hyposialylated Angptl4 in podocytes and its susceptibility to treatment with sialic acid precursors (Clement et al., 2011; Chugh et al., 2012). Sialylation of proteins is a common event in human cells, and sialic acid is most commonly incorporated at *O-* and *N-* glycosylation sites of glycoproteins, and in glycosphingolipids (gangliosides). It is important to understand the differences between structural and secreted proteins in terms of their requirement for sialic acid. A substantial amount of sialic acid in cells is recycled (**Figure 3**), which reduces tremendously the burden for *de novo* sialic acid synthesis (Bertozzi et al., 2009). This recycled sialic acid likely comes mostly from structural, and to a lesser extent, endocytosed proteins, since sialylation of secreted proteins represents a net loss of total cellular sialic acid content. This net loss must then be made up by *de novo* sialic acid synthesis. Humans synthesize sialic acid from glucose (**Figure 3**), since there is no major nutritional source of *N*-acetylneuraminic acid (NANA/Neu5Ac), the predominant form of sialic acid present in humans (Varki and Schauer, 2009). Animals convert NANA into *N*-glycolylneuraminic acid (NGNA/Neu5Gc) using the enzyme NANA hydroxylase, which is non-functional in humans due to an exon deletion/frame shift mutation (Chou et al., 1998). Under most circumstances, NGNA is not incorporated into human proteins, thereby excluding diet as a source of sialic acid in humans. Some organs, like liver and adipose tissue, that produce large number of secreted proteins have a very active sialic acid biosynthesis pathway, and therefore, in the current context, secrete sialylated Angptl4 into the circulation normally and during times of high demand (e.g., fasting, nephrotic syndrome). Podocytes likely have a less active sialic acid biosynthesis pathway (Chugh et al., 2012), and in the setting of severe upregulation of Angptl4 in MCD and diabetic nephropathy, this pathway is unable to cope with high demand, despite a slight increase in the expression of the rate limiting enzyme GlcNAc 2-epimerase (GNE) in both disorders. This results in the secretion of a combination of hyposialylated and sialylated Angptl4 from podocytes. An additional factor in this equation in podocytes may be limited activity of a class of enzymes that transfers sialic acids to glycoproteins, called sialyltransferases.

The choice of ManNAc over other potential agents, including purified sialic acid, for sialylation based therapeutics was determined by several factors (Chugh et al., 2012). Unlike NANA, ManNAc has a neutral charge, which allows it to cross cell membranes easily. Moreover, ManNAc is a naturally occurring precursor molecule and is not incorporated directly at sialylation sites, whereas NANA is the end product, and raising blood levels of NANA to increase cellular uptake could theoretically affect function of endothelial cell sialylated transmembrane proteins. High plasma sialic acid levels have been noted in a variety of disease states (Sillanaukee et al., 1999), but causality has not been established. By contrast, ManNAc gets converted to NANA inside cells where it is utilized for protein sialylation. Exogenously administered ManNAc enter the sialic acid biosynthesis pathway after the rate limiting step catalyzed by UDP–GNE (**Figure 3**), thereby surmounting the intrinsic limitations of the pathway during high demand states. Diseases involving the podocyte are especially suited for ManNAc therapy for two reasons. Unlike most other cells in the body, podocytes divide very infrequently, which sets the stage for accumulation in these cells and consequently lower oral dose requirement when compared to therapy targeting dividing cells. Rapid urinary excretion after oral dosing (90% loss of oral dose in urine by 4 h; Malicdan et al., 2009) is also an advantage, since podocytes are exposed to most of the oral dose while being filtered through the glomerulus. A combination of low dose requirement for podocyte disease and rapid urinary excretion reduces overall chances of systemic toxicity.

Based on our current experience, we would consider using ManNAc for maintenance therapy in both diabetic nephropathy and MCD, either as a daily very low dose regimen, or intermittent low dose therapy. Even though long term glucocorticoid therapy is associated with multi-organ complications, the first episode of MCD should still be treated with glucocorticoids, especially in children, at least in part to prove that the disease is indeed glucocorticoid sensitive. Also, glucocorticoids reduce podocyte Angptl4 upregulation (Clement et al., 2011) and increase Angptl4 secretion from skeletal muscle, heart and adipose tissue (discussed later), both of which benefit patients with MCD. Since 80–90% of children relapse after the first remission (Koskimies et al., 1982; Tarshish et al., 1997), ManNAc is perfectly suited as a maintenance drug to reduce the frequency and intensity of relapse. Glucocorticoids are not used in the treatment of diabetic nephropathy, since they aggravate the underlying diabetic state.

## RECOMBINANT ANGPTL4 MUTANTS FOR TREATMENT OF DIABETIC NEPHROPATHY AND FSGS

A recent study published by our group shows how circulating Angptl4 is an intrinsic component of nephrotic syndrome, and functions in tandem with plasma albumin and free fatty acids (FFAs) to link proteinuria with hypertriglyceridemia (Clement et al., 2014). Albumin is the most abundant plasma protein that circulates as a 69 kDa monomer, and serves as a vehicle to transport cations, hormones, and FFA. FFA are a critical energy source for the body, and also serve as an important molecular mediator in nephrotic syndrome. Organs like skeletal muscle and heart utilize FFA for energy (Mattijssen and Kersten, 2012), while adipose tissue recycles FFA, since it both releases and takes up FFA for storage as triglycerides. Normal sources of fatty acids include diet, mobilization from adipose tissue, and conversion of excess carbohydrates into fat by the liver. Some fatty acids are coupled with glycerol to form triglycerides (or triacylglycerols) for transport or storage, and can be converted back into FFA by lipases. The rest circulate in blood as FFA, mostly coupled non-covalently with albumin. Albumin molecules have six high affinity FFA binding sites, and many low affinity binding sites, and up to ten FFA molecules can be bound to an albumin molecule at any given time (Papamandjaris et al., 1998). Adipose tissue releases FFA into circulation after serial conversion of triglycerides to diglycerides by adipose triglyceride lipase, and diglycerides to monoglycerides by hormone sensitive lipase. After digestion of dietary fat, medium chain fatty acids (8–12 carbon chain) are transported coupled with albumin, whereas long chain fatty acids (14 or more carbon chain) are converted back to triglycerides, incorporated into chylomicrons, and transferred to the circulation via the thoracic duct.

**FIGURE 4:**
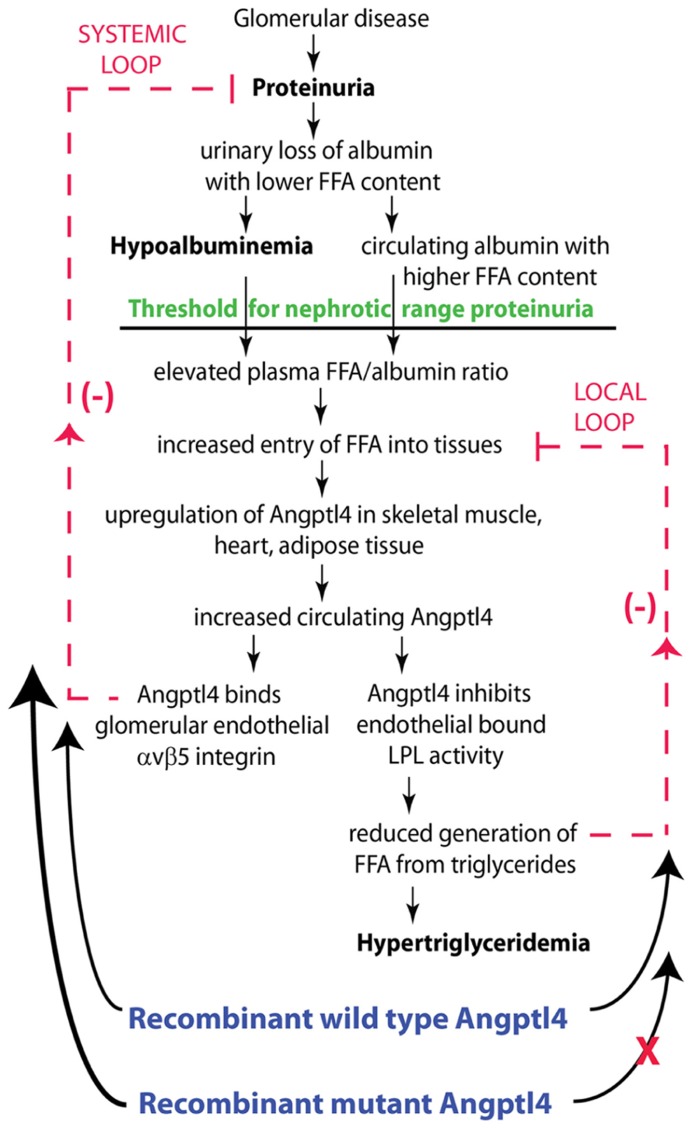
**Schematic illustration of negative feedback loops in the link between proteinuria, hypoalbuminemia and hypertriglyceridemia mediated by Angptl4 and FFA (free fatty acids).** Plasma FFA are non-covalently bound to albumin, and because of the preferential loss of albumin with low FFA content during proteinuria, albumin with higher FFA content is retained in circulation. As glomerular disease progresses and proteinuria increases, hypoalbuminemia develops, and the combination of high albumin FFA content and lower plasma albumin levels increases the plasma FFA/albumin ratio. This increased available FFA enters skeletal muscle, heart and adipose tissue to induce upregulation of Angptl4, at least in part mediated by PPARs. Angptl4 secreted from these organs participates in two feedback loops. In the systemic loop, it binds to glomerular endothelial α_v_β_5_ integrin and reduces proteinuria, the principal driver of nephrotic syndrome. In a local loop, it inhibits LPL activity in the same organs from which it is secreted to reduce the uptake of FFA, thereby curtailing the stimulus for its own upregulation. Exogenously administered recombinant wild type Angptl4 affects both loops, similar to endogenous Angptl4, thereby reducing proteinuria and increasing plasma triglyceride levels. Recombinant mutant forms of Angptl4 bypass the local loop, and act only on the systemic loop, thereby reducing proteinuria without raising plasma triglyceride levels. Adapted from Clement et al. (2014).

Sources of FFA for uptake by organs includes albumin bound FFA, and conversion of circulating triglycerides into FFA by the endothelium anchored enzyme LPL. In nephrotic syndrome, the balance between these two sources of FFA uptake is significantly altered. Proteinuric kidneys preferentially loose albumin with a low FFA content, resulting in progressive retention of albumin with high FFA content (Ghiggeri et al., 1987; Clement et al., 2014) (**Figure 4**). As proteinuria reaches nephrotic range, hypoalbuminemia develops, and a combination of high FFA containing albumin and hypoalbuminemia raises the plasma ratio of FFA to albumin (Clement et al., 2014). This elevated plasma FFA to albumin ratio drives increased FFA uptake in skeletal muscle, heart and adipose tissue, which in turn increases Angptl4 expression and secretion from these tissues. Since Angptl4 is a known peroxisome proliferator-activated receptor (PPAR) target gene (Kersten et al., 2009; Staiger et al., 2009; Georgiadi et al., 2010), and PPAR expression is increased during the nephrotic phase in these tissues (Clement et al., 2014), at least part of this Angptl4 upregulation is likely to be PPAR mediated. Angptl4 secreted from these organs into the circulation has two effects. First, it binds to the αvβ5 integrin in glomerular endothelium and reduces proteinuria (**Figure 4**, systemic feedback loop). The precise mechanism by which the Angptl4 – αvβ5 integrin interaction reduces proteinuria is not known, but it is possible that additional feedback loops within the glomerulus are involved. Second, Angptl4 inactivates LPL activity in these organs, thereby reducing the conversion of triglycerides into FFA, which reduces FFA uptake by this pathway (**Figure 4**, local feedback loop), and also results in hypertriglyceridemia. In summary, the retention of FFA in nephrotic syndrome tends to accelerate production of Angptl4 from peripheral organs, whereas its own effect on LPL activity in these same organs limits the extent of upregulation (Clement et al., 2014).

In many ways, this attempt to reduce proteinuria by Angptl4 represents a systemic response against rising proteinuria. The local feedback loop slows down this anti-proteinuric effect of circulating Angptl4. Since this is a naturally occurring response in the body, these pathways could be manipulated pharmacologically to attain a prolonged anti-proteinuric effect in one of two ways. First, existing pharmacological agents could be used to increase the intrinsic production of Angptl4 from skeletal muscle, heart and adipose tissue. PPAR agonists (Mattijssen and Kersten, 2012), β2-adrenergic receptor agonists (Mattijssen and Kersten, 2012), glucocorticoids (Koliwad et al., 2009), and FFA supplementation (Clement et al., 2014) increase Angptl4 expression in these organs. This may contribute to the partial efficacy of glucocorticoids in different diseases that cause nephrotic syndrome, since they are a component of most existing treatment regimens. This approach, however, has limitations, since increasing intrinsic Angptl4 production in these organs will activate both systemic and local feedback loops, worsen hypertriglyceridemia and potentially reduce the uptake of FFA, a major source of energy, into skeletal muscle and heart, causing end organ dysfunction. Moreover, PPAR agonists and glucocorticoids have very significant toxicities due to effects on multiple pathways.

**FIGURE 5:**
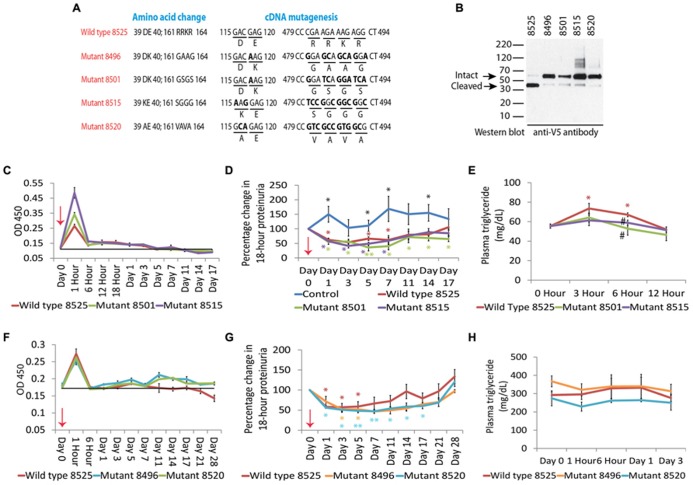
**(A)** Schematic representation of wild type and mutant human Angptl4 proteins showing mutations in areas important for LPL binding (amino acid 40, and adjacent amino acid 39) and protein cleavage (amino acids 161 to 164). **(B)** Western blot of recombinant tagged proteins using mouse anti V5 antibody to demonstrate the expected size of the intact protein and reduced cleavage in the mutant proteins (arrows). **(C)** Plasma levels of wild type (8525) or mutant (8501, 8515) human Angptl4 in Buffalo Mna rats (*n* = 3 rats/group) after injecting 55 μg of recombinant human protein as assessed by OD 450 using reagents from the human Angptl4 ELISA kit. **(D)** Effect of injecting wild type and mutant Angptl4 on proteinuria in Buffalo Mna rats. **(E)** Effect of injecting wild type and mutant Angptl4 on plasma triglyceride levels in Buffalo Mna rats. **(F)** Plasma levels of wild type (8525) and mutant (8496, 8520) human Angptl4 proteins as assessed by OD 450 after injecting a lower dose (15 μg) in Zucker Diabetic Fatty (ZDF) rats (*n* = 4 rats/group). **(G)** Effect of injecting wild type and mutant Angptl4 on proteinuria in ZDF rats. **(H)** Effect of 15 μg of wild type and mutant Angptl4 on plasma triglyceride levels in ZDF rats. Error bars are s.e.m. *t*-test, two way, *P < 0.05, **P < 0.01. In panel **D**, black * are shown where all three study groups were individually different from control injected rats. In panels **D**, e.g., colored * shown where individual values were significantly different from corresponding baseline values. # in panel **E** is *P* < 0.05 in mutant protein groups compared to wild type Angptl4 injected rats. Adapted from Clement et al. (2014).

Another way to manipulate this naturally occurring pathway is to use recombinant human Angptl4, which would be superior to the agents mentioned above, since both therapeutic and side effects will be selective for this protein (**Figure 4**). Since native recombinant human Angptl4 in circulation will have the same limitations as tissue secreted Angptl4, it is appropriate to modify the protein to ignore the local feedback loop (“the brake”), while acting solely on the systemic feedback loop (“the accelerator”). This would allow for achieving higher circulating Angptl4 levels than otherwise possible by using existing agents, without incurring the risk of hypertriglyceridemia or starving skeletal muscle and heart of energy substrate. Since population based studies (Romeo et al., 2007; Romeo et al., 2009) show that individuals carrying the E40K variant have very low triglyceride levels and reduced ability of Angptl4 to inhibit LPL *in vitro* (Yin et al., 2009), we developed recombinant mutant forms of human Angptl4 with changes at amino acid 40 or its neighbor amino acid 39 (**Figure 5**). Also, since Angptl4 readily cleaves between amino acids 161 to 164, we made additional changes in this region to improve the half-life of the intact protein. Studies with recombinant rat Angptl4 have shown the formation of very high order oligomers that migrate even slower than α2-macroglobulin (720 kDa) on one-dimensional non-reducing gels (Clement et al., 2011). This feature, combined with the ability of Angptl4 to bind HDL particles in circulation (Mandard et al., 2006), helps prolong its half-life in circulation in the setting of nephrotic syndrome, in which non-oligomerizing proteins like albumin and IgG are readily lost in urine. Wild type human and six different Angptl4 mutant clones and their HEK 293 based stable cell lines were developed and tested (Chugh, 2011a). Published work on the wild type and four of these mutants (Clement et al., 2014) (**Figures 5A–H**), including pharmacokinetic studies in nephrotic rats, show that mutagenesis did not reduce the ability to reduce proteinuria in Buffalo Mna rats (a model of FSGS) and Zucker diabetic fatty rats (ZDF, a model of diabetic nephropathy). Some of the mutant proteins reduced proteinuria more substantially and for a longer duration than the wild type protein (**Figures 5D,G**). Plasma triglyceride levels increased with the wild type, but not the mutant proteins. A single 55 μg dose of recombinant human Angptl4 reduced proteinuria for over 2 weeks, with peak reduction of up to 65% in Buffalo Mna rats (**Figure 5D**). Finally, a smaller dose (15 μg) of wild type recombinant Angptl4 in ZDF rats reduced proteinuria without causing hypertriglyceridemia (**Figure 5G**), suggesting a lower threshold for Angptl4 to act via the systemic loop, compared to the local feedback loop. These recombinant proteins are now being produced on a large scale to allow for expanded testing and prolonged administration in nephrotic rats to study effects on chronic kidney disease. Since Angptl4 is a normal circulating protein with levels that vary several fold with fasting / feeding, the likelihood of major aberrant side effects of therapy with mutant Angptl4 protein is relatively low. However, like any mutated protein used for therapeutics, there is a potential for developing an antibody response in some patients that may limit long term usage in these patients.

In summary, sialylation based and recombinant mutated Angptl4 based therapeutic strategies hold significant promise in the treatment of common forms of proteinuric chronic kidney disease, including diabetic nephropathy. Both areas are novel, protected by intellectual property, innovative, mechanism based, and have been extensively studied *in vivo* in appropriate human disease models. Moreover, the central role played by Angptl4 in nephrotic syndrome (at par in importance with albumin, FFA and triglycerides) suggests that manipulating Angptl4 related pathways in the context of therapeutics has a high chance of success.

## PERSPECTIVE ON FUTURE DEVELOPMENT OF ADDITIONAL NOVEL THERAPIES

The discovery of Angptl4 as a major player in human nephrotic syndrome was based on a strategy to identify and selectively investigate genes/ proteins that could potentially link at least two of the three major components (proteinuria, hyperlipidemia, and edema) of nephrotic syndrome. This approach continues to have potential in the future, since the pathogenesis of hypercholesterolemia, proteinuria, and increase peripheral capillary permeability related to edema are not completely understood. Also, the reason for the muted response of the liver to counter hypoalbuminemia in nephrotic syndrome by increasing albumin synthesis in most patients is unclear. In developing future therapies, it is important to steer clear of old unyielding hypotheses, like Shalhoub’s hypothesis of putative T-cell secreted factors as the causal mediator of MCD, or the charge hypothesis of glomerular permeability to explain selective proteinuria. Investigators must classify early footprints of glomerular disease based on molecular changes, rather than current morphology based standards. In this context, selective proteinuria could be better explained by looking for uniformity of early molecular changes in the glomerulus in MCD, whereas non-selective proteinuria potentially attributable to presence on non-uniform early changes in other diseases. Unless an objective and goal directed approach is adopted toward developing modern therapeutics, future nephrologists may have to continue to adapt drugs developed for non-kidney indications to treat kidney disease.

## Conflict of Interest Statement

Sumant S. Chugh is Founder, President and Chief Executive Officer of GDTHERAPYLLC, and filed patents related to the use of Angpt4 mutants (PCT/US2011/039255) and precursors of sialic acid, including ManNAc (PCT/US2011/039058) for the treatment of nephrotic syndrome. SSC may benefit financially from these patents in the future. None of the other authors declared competing financial interests.
